# Supplementary material for: Cryo-EM structure of the Spo11 core complex bound to DNA
Source: bioRxiv. 2023 Nov 1:2023.10.31.564985. Preprint. [Version 1] doi: 10.1101/2023.10.31.564985 (PMC10634984; doi:10.1101/2023.10.31.564985)
Supplement: Supplement 1 [file media-1.pdf]

Supplemental information for:

Cryo-EM structure of the Spo11 core complex bound to DNA

***You Yu[1], *Juncheng Wang[1], *Kaixian Liu[2], *Zhi Zheng[2,3], Meret Arter[2], Corentin Claeys Bouuaert[2,4], Stephen Pu[2,4], †Dinshaw J. Patel[1,3] and †Scott Keeney[2,3,4]**

[1]Structural Biology Program, Memorial Sloan Kettering Cancer Center, New York, NY 10065
[2]Molecular Biology Program, Memorial Sloan Kettering Cancer Center, New York, NY 10065
[3]Louis V. Gerstner Graduate School of Biomedical Sciences, Memorial Sloan Kettering Cancer Center, New York, NY 10065
[4]Howard Hughes Medical Institute, Memorial Sloan Kettering Cancer Center, New York, NY 10065

*Equal contribution

†Correspondence to DJP (pateld@mskcc.org) and SK (s-keeney@ski.mskcc.org)

This pdf contains:

Supplemental Figures S1-S9

Supplemental Tables S1-S3

SUPPLEMENTAL FIGURES

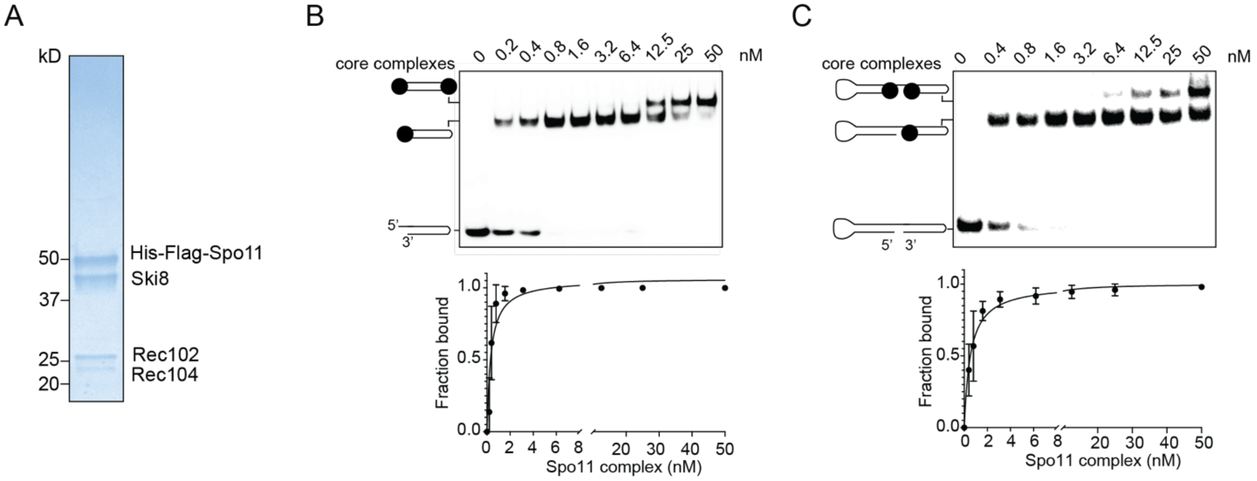

Fig. S1. Spo11 core complex purification and EMSA assays.
(A) SDS-PAGE gel of purified core complex (~1 µg), stained with Coomassie.
(B,C) EMSA assays and quantification of core complex binding to the hairpin DNA substrate (B) and the gapped DNA substrate (C). In panel C, the position of the second core complex that gives the slowest migrating band is unknown; it could be at the nick location as shown or it could associate with one of the hairpin ends. Error bars indicate mean ± SD of three replicates.

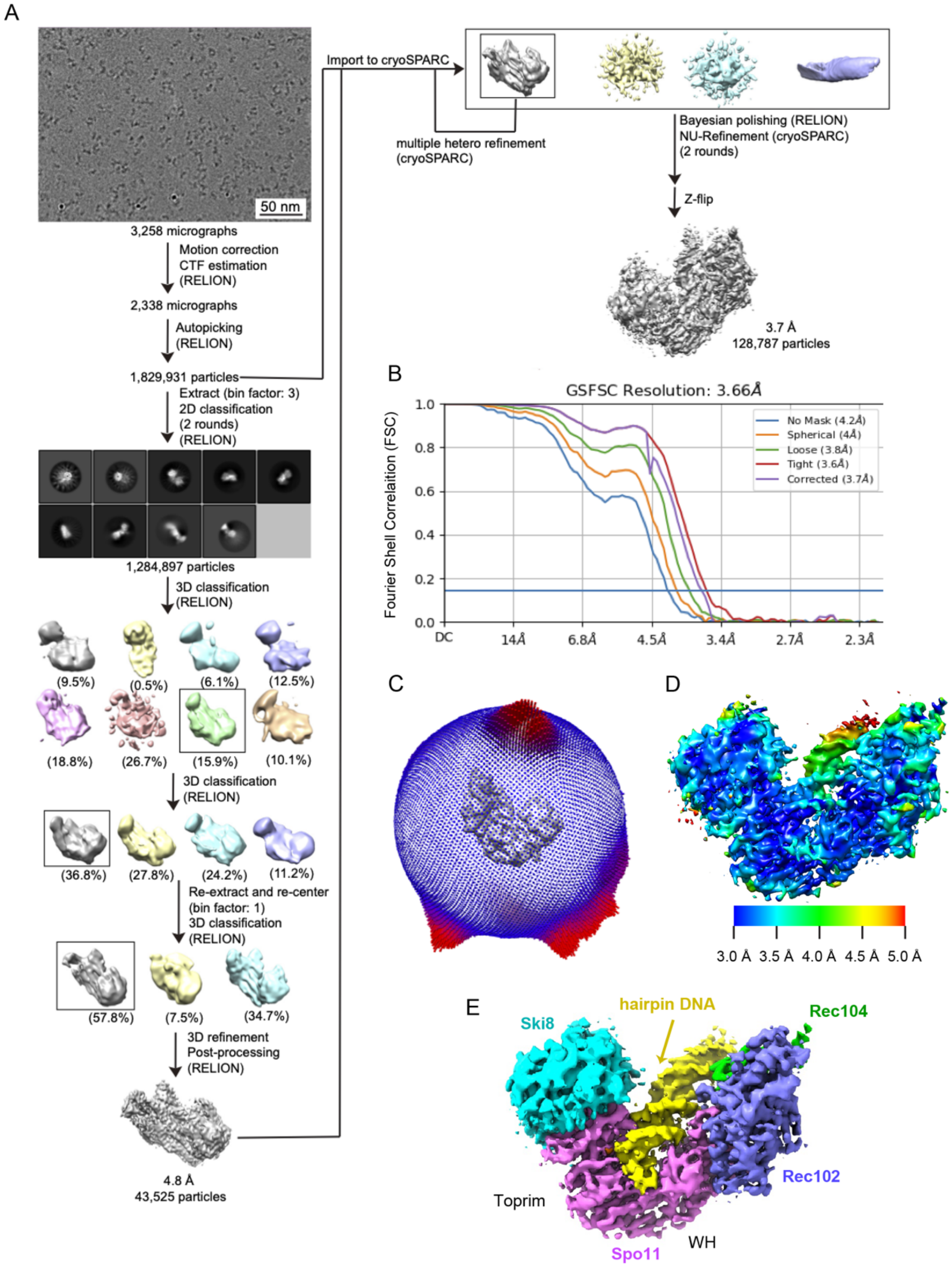

Fig. S2. Cryo-EM reconstruction of the core complex bound to hairpin DNA.
(A) Flow chart of cryo-EM image processing.
(B) Global Fourier shell correlation (FSC) curves. The overall cryo-EM map resolution is 3.7 Å with FSC set at 0.143.
(C, D) Euler angle distribution (panel C) and final 3D reconstructed map colored according to local resolution (panel D).
(E) Density map.

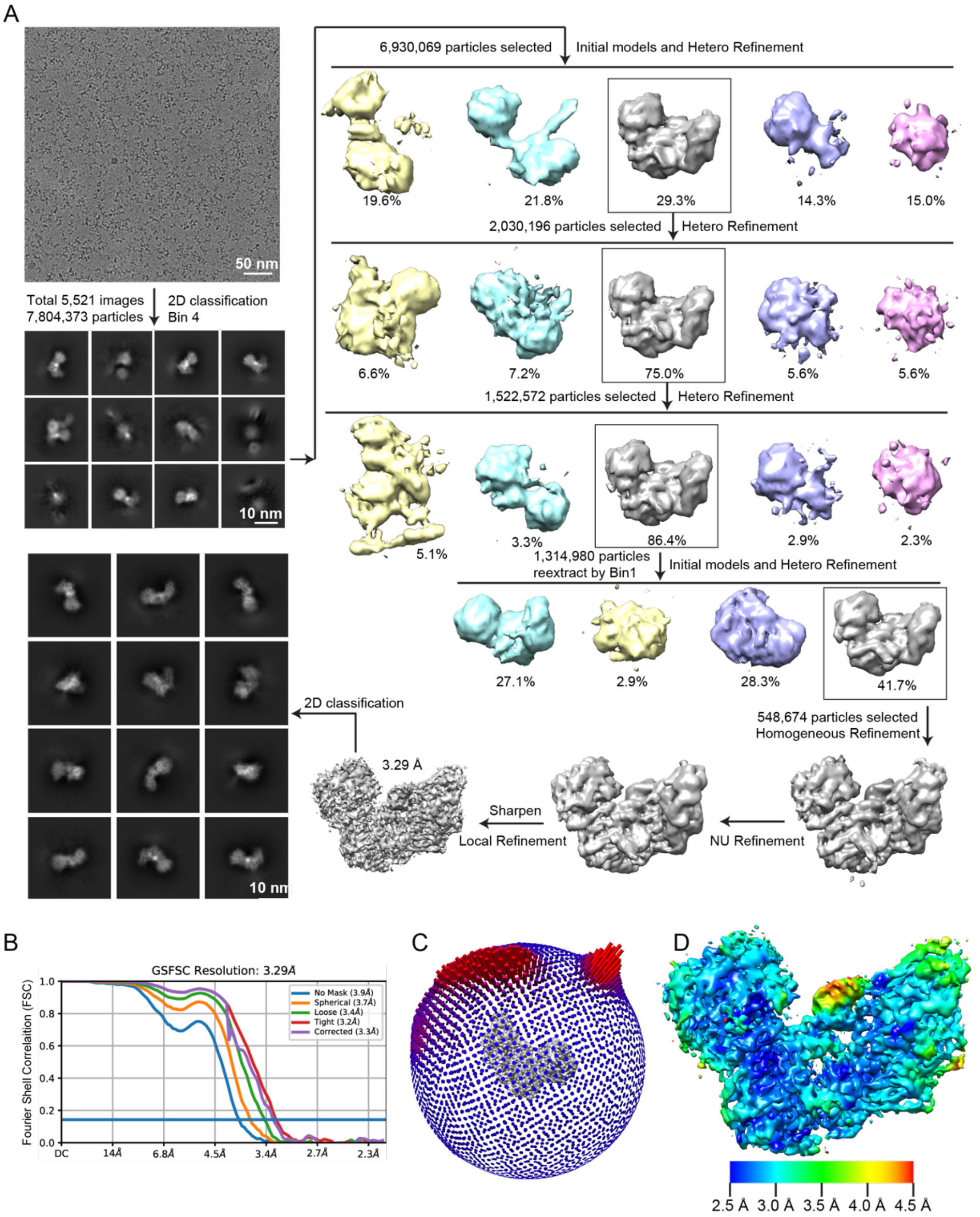

Fig. S3. Cryo-EM reconstruction of the core complex bound to gapped DNA.
(A) Flow chart of cryo-EM image processing.
(B) Global FSC curves. The overall cryo-EM map resolution is 3.3 Å with FSC set at 0.143.
(C, D) Euler angle distribution (panel C) and final 3D reconstructed map colored according to local resolution (panel D).

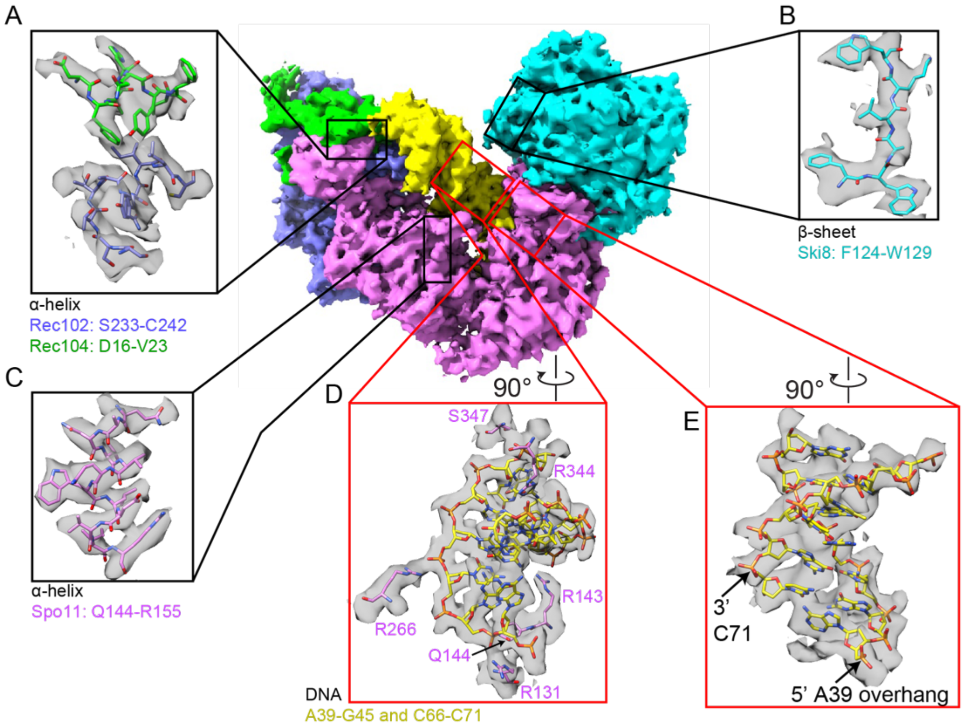

Fig. S4. Examples of protein side chain and DNA base-sugar-phosphate identification in the cryo-EM structure of the core complex bound to gapped DNA.

(A–E) The expanded boxes show examples of fitting of protein amino acid side chain (panels A–C) and DNA base-sugar-phosphate and interacting protein side chains (panels D and E) into the density map.

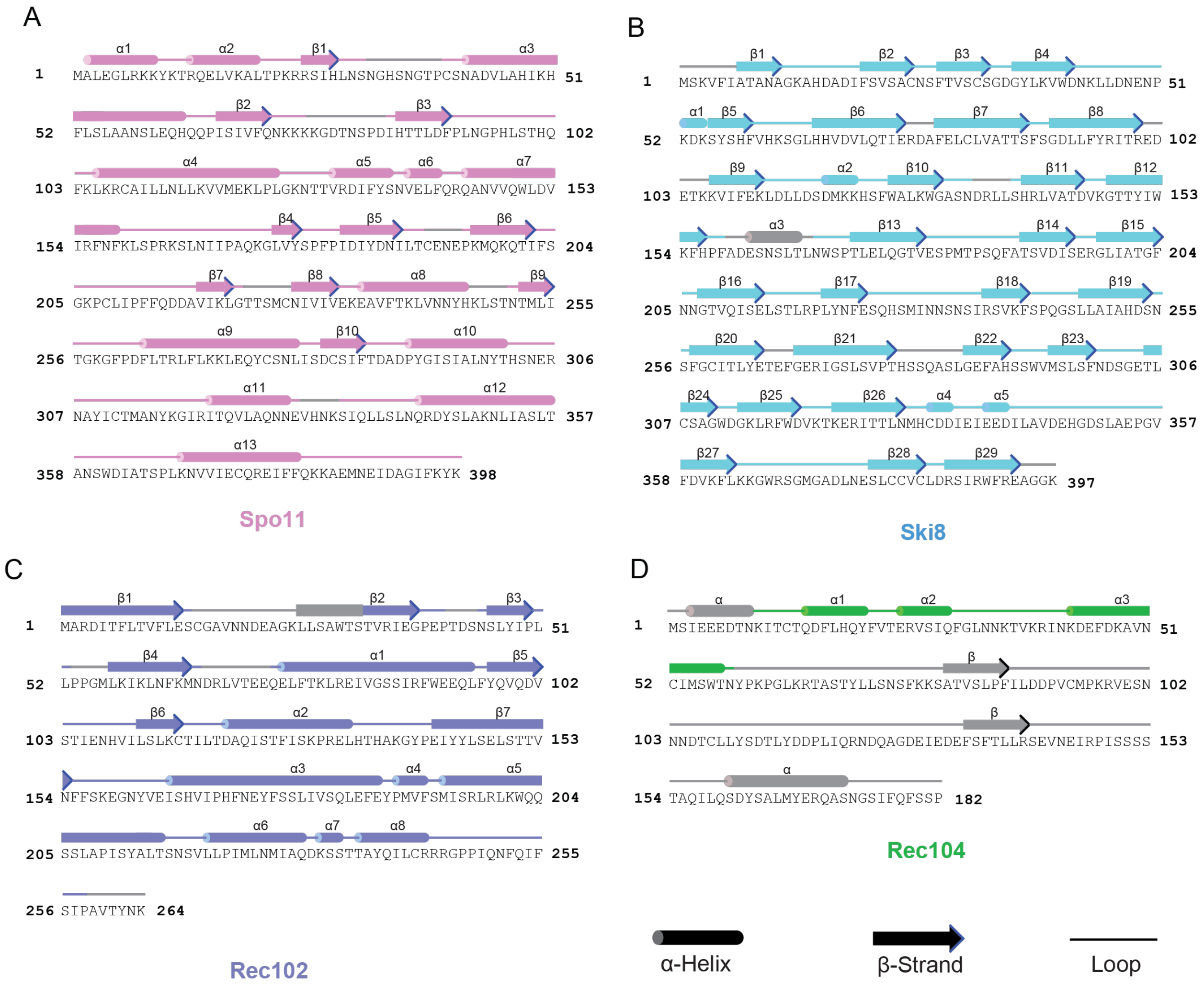

Fig. S5. Protein secondary structures.
(A–D) The secondary structures for Spo11 (panel A), Ski8 (panel B), Rec102 (panel C), and Rec104 (panel D) were determined from the cryo-EM structure of the core complex bound to gapped DNA. Gray-colored regions are not visible in the cryo-EM structure.

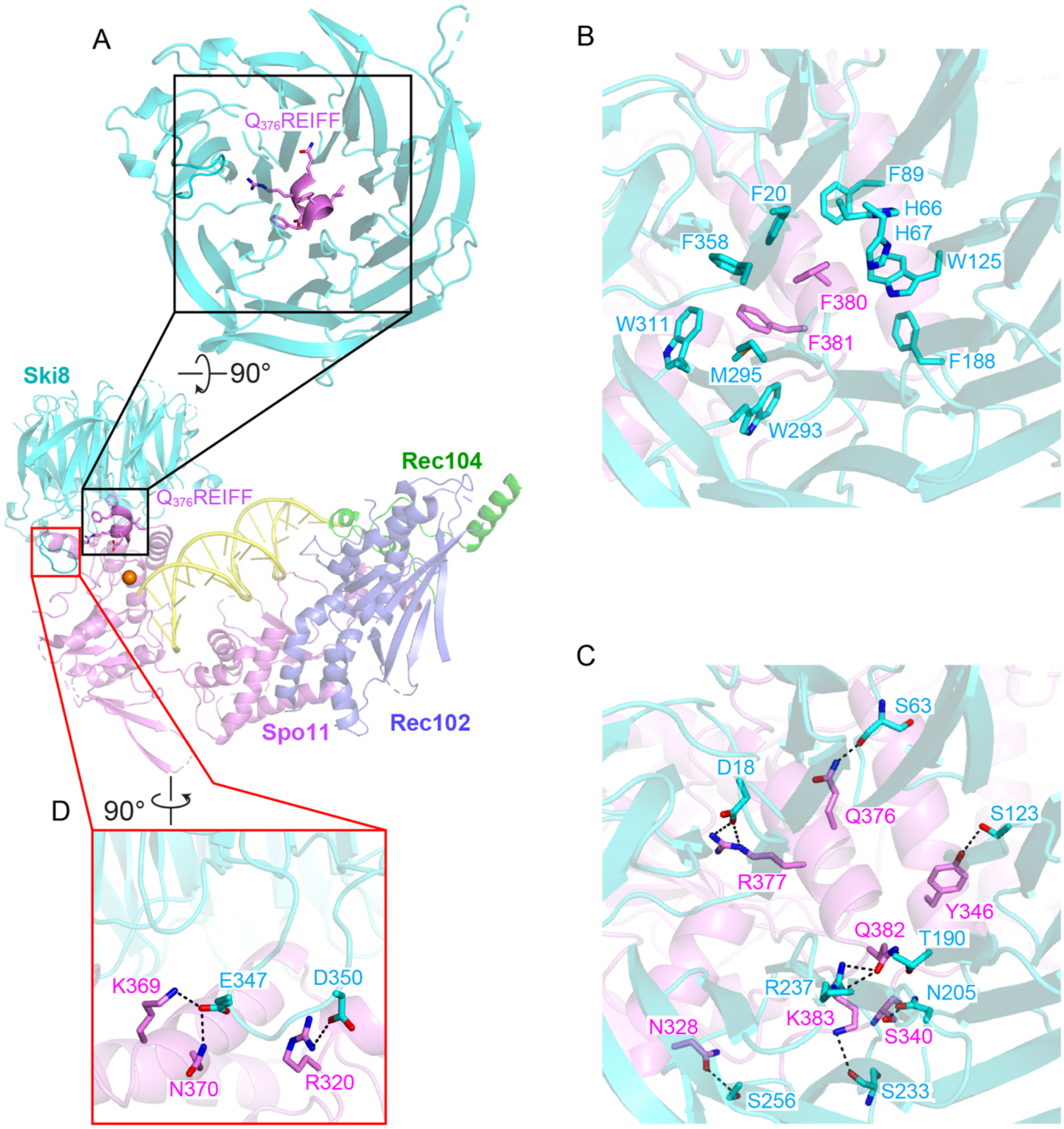

Fig. S6. Interactions between Ski8 and Spo11.

All images are from the cryo-EM structure of the core complex bound to gapped DNA.

(A) QREIFF motif from Spo11 (magenta) and the WD40 repeats from Ski8 (cyan).

(B) Hydrophobic interactions between Ski8 WD40 repeats and Spo11.

(C) Hydrogen bond interactions between Ski8 and Spo11.

(D) Additional hydrogen bonding interactions between Spo11 and the extended loop in Ski8.

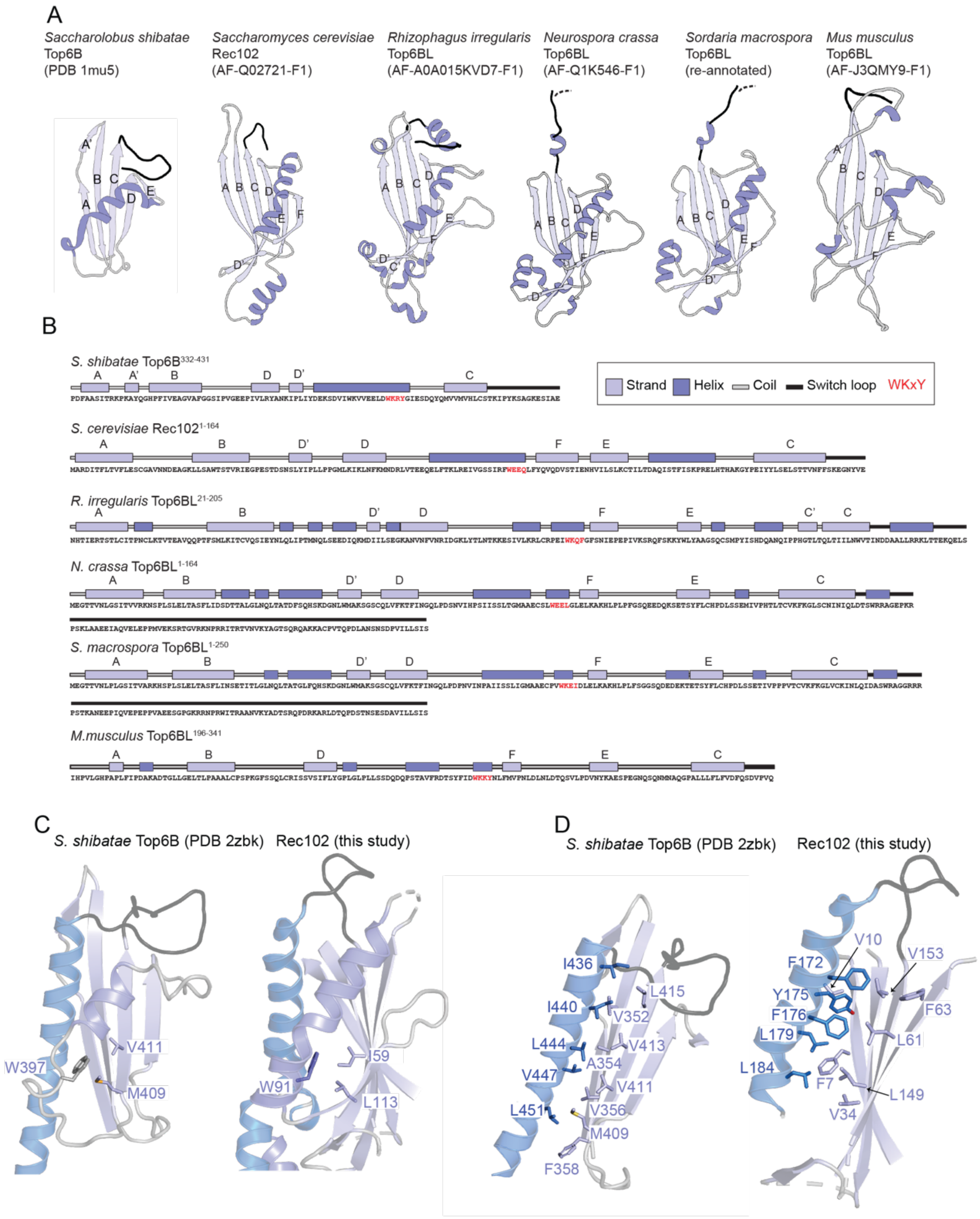

Fig. S7. Sequence and structural conservation of Rec102 and Top6B.

(A) Top6B and Rec102/Top6BL architectures. Strands, helices, and switch loops are colored as in **Fig. 3B** and strands are labeled as in **Fig. 3A**. All structures show truncated proteins, starting at the first β strand (strand A) and ending with the switch loop before the start of the stalk. The switch loop is truncated for the *N. crassa* and *S. macrospora* proteins for clarity.

(B) Secondary structure and sequence information for models shown in (A). The conserved WKxY motif is highlighted in red and β strands are labeled as in (A).

(C) Packing for *S. shibatae* Top6B (PDB: 2zbk) in comparison to Rec102 for the W in the WKxY motif. Ribbons and sticks are colored as in **Fig. 3B**.
(D) Detail view of the hydrophobic interface between the amphipathic stalk and the β strands; related residues in Top6B and Rec102 are indicated.

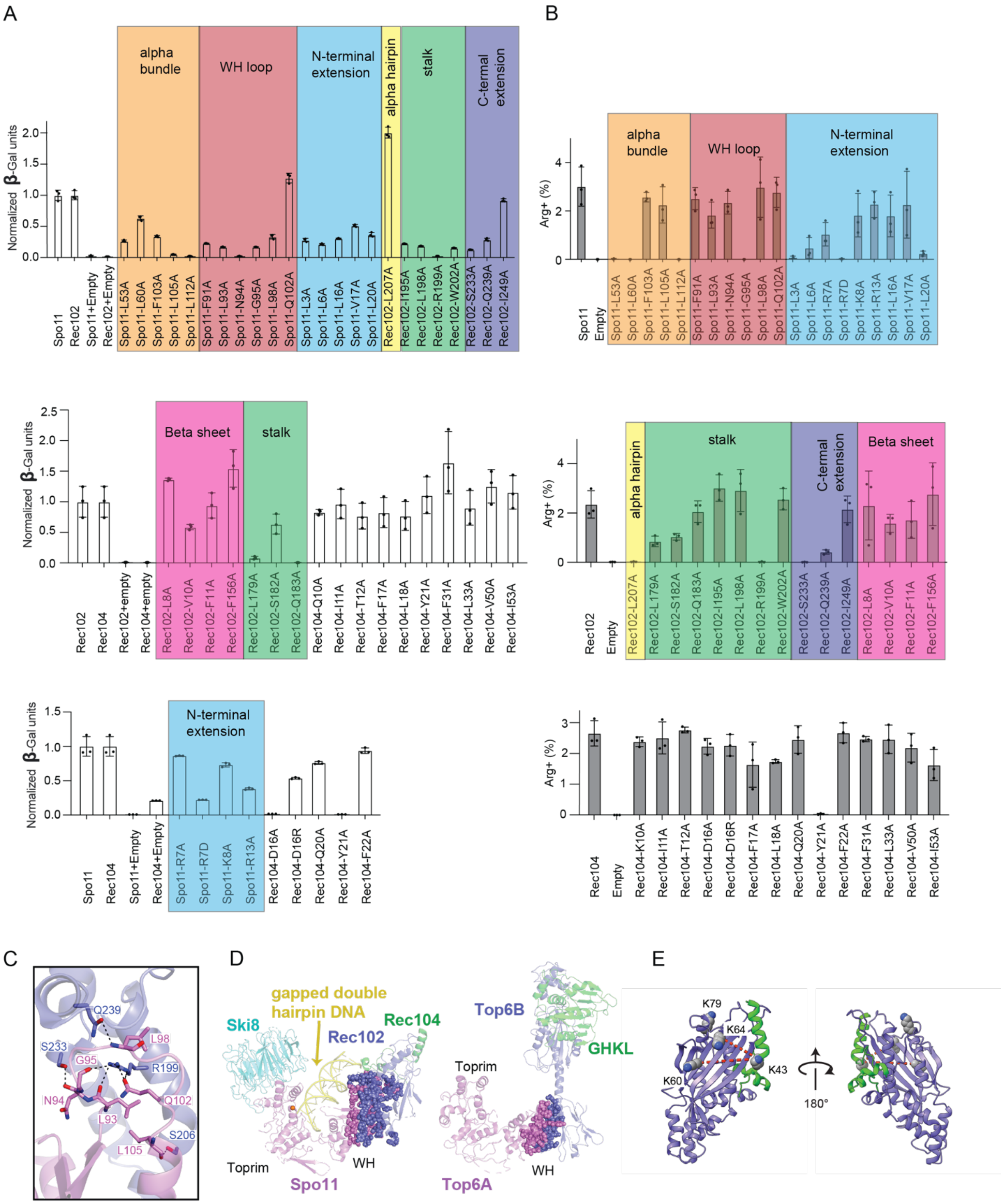

Fig. S8. Functional analyses of protein-protein interfaces within the core complex.
(A) Quantitative β-galactosidase assays to measure Y2H interactions of mutants of GAL4AD-Spo11 and LexA-Rec102 (top), LexA-Rec102 and GAL4AD-Rec104 (middle) and GAL4AD-

Spo11 and Rec104-LexA (bottom). The interaction of Rec102 with Rec104 was measured in vegetative conditions and the rest were measured in meiotic conditions.
(B) Heteroallele recombination assays with different Spo11 (top), Rec102 (middle) and Rec104 (bottom) mutant proteins. The graphs show the frequency of Arg+ prototrophs generated by recombination between *arg4-bgl* and *arg4-nsp* alleles[54]. In panels A and B, bars show mean ± SD of three replicates; points show individual measurements.
(C) Hydrogen bonding interactions between Spo11 and Rec102.
(D) Comparison of interfacial areas for Spo11 with Rec102 (1,680 $Å^2$, complex with gapped DNA) vs. Top6A with Top6B from *M. mazei* Topo VI (958 $Å^2$, PDB: 2q2e). Residues involved in the interfaces are shown as spheres.
(E) Positions of lysines in Rec102 and Rec104 that are readily crosslinked. Red dashed lines connect the α carbons of lysines that were crosslinked in[14].

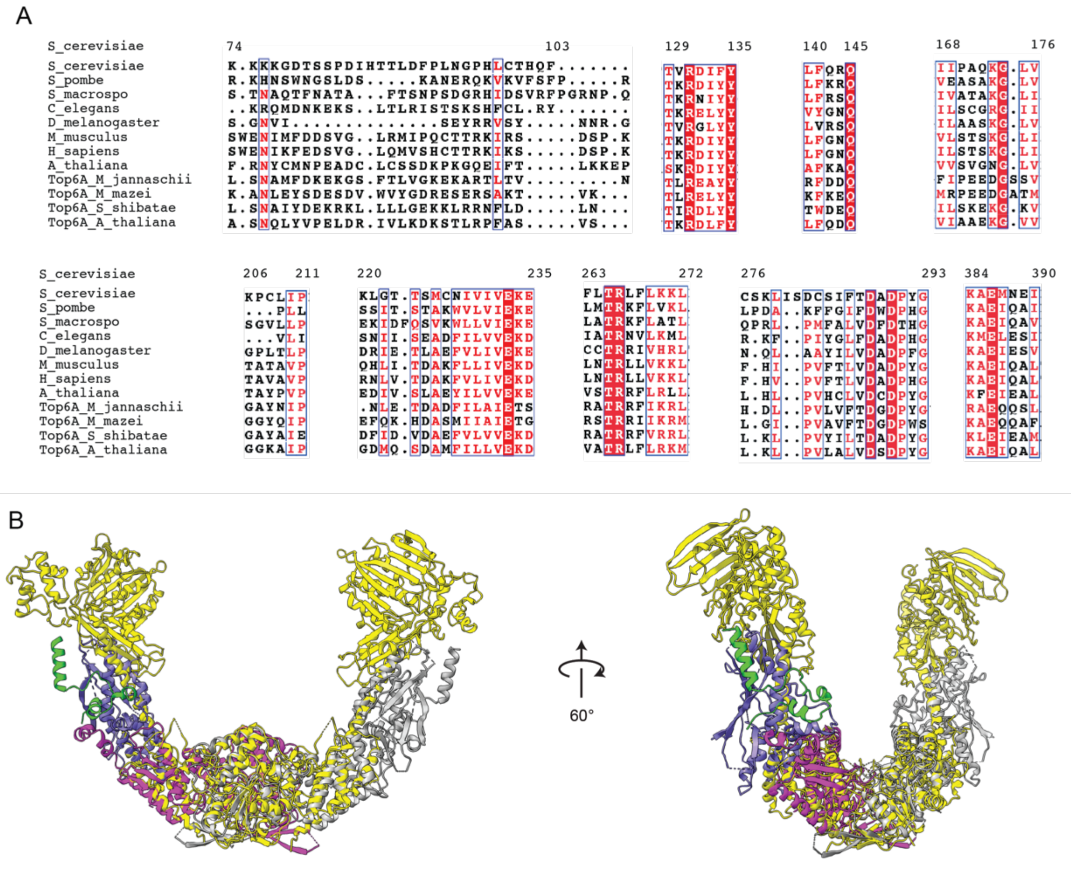

Fig. S9. Spo11 sequence conservation.
(A) Multiple sequence alignment of Spo11 and Top6A orthologs at protein-protein and protein-DNA contact sites.
(B) Structural alignment of the hypothetical model of a Spo11 core complex pre-DSB dimer and the crystal structure of the dimeric *S. shibatae* Topo VI holoenzyme (yellow). One copy of the Spo11 core complex is colored as **Fig. 1B**, and the other copy is colored in gray. Ski8 is not shown for simplicity.

SUPPLEMENTAL TABLES

Table S1. Cryo-EM data collection, processing, and validation statistics

	Spo11 core complex with:	
	Hairpin DNA	Gapped DNA
Data collection		
Microscope	Titan Krios	Titan Krios
Detector	Gatan K3	Gatan K3
Automation software	SerialEM	SerialEM
Nominal magnification	22,500	22,500
Voltage (kV)	300 kV	300 kV
Total dose (e^-/Å^2)	53.00	53.00
Dose rate (e^-/pixel/s)	20	20
Number of frames collected	40	40
Defocus range (μm)	-1.0 to -2.5	-1.0 to -2.5
Pixel size (Å)	1.064	1.064
Collected Micrographs	3,258	5,521
Selected Micrographs	2,388	5,221
Reconstruction		
Initially autopicked particles	1,829,931	7,804,373
Particles used for classification	1,284,897	6,930,069
Particles in the final map	128,787	548,674
Symmetry	C1	C1
Resolution		
FSC 0.5 (unmasked/masked, Å)	4.1/3.9	3.7/3.6
FSC 0.143 (unmasked/masked, Å)	3.7/3.6	3.3/3.3
Map sharpening B factor (Å^2)	-158.0	-155.8
Model composition		
Protein residues	1001	993
Nucleotide	43	31
Nonhydrogen atoms	8895	8569
Validation		
MolProbity	2.80	2.84
Clash score	20	18
Map Correlation Coefficient	0.74	0.71
R.m.s. deviations		
Bond lengths (Å)	0.005	0.017
Bond angles (°)	0.979	1.250
Ramachandran plots		
Favored (%)	88.11	88.88
Allowed (%)	10.95	10.81
Outliers (%)	0.94	0.31
Rotamer outliers (%)	3.21	4.61

Table S2. Yeast strains

Strain	Genotype
SKY661	*MATa, ho::LYS2, lys2, leu2::hisG, trp1::hisG, ndt80::kanMX, LexA(op)-LacZ::URA3*
SKY662	*MATα, ho::LYS2, lys2, leu2::hisG, trp1::hisG, ndt80::kanMX, LexA(op)-LacZ::URA3*
SKY1311	*MATα, ho::LYS2, lys2, ura3, leu2::hisG, trp1::hisG, arg4-nsp, rec102∆::URA3*
SKY1312	*MATa, ho::LYS2, lys2, ura3, leu2::hisG, trp1::hisG, arg4-bgl, rec102∆::URA3*
SKY969	*MATα, ho::LYS2, lys2, leu2::hisG, trp1::hisG, arg4-bgl, spo11Δ::hisG-URA3-hisG*
SKY970	*MATa, ho::LYS2, lys2, leu2::hisG, trp1::hisG, arg4-nsp, spo11Δ::hisG-URA3-hisG*
SKY7404	*MATa, ho::LYS2, lys2, leu2::hisG, trp1::hisG, arg4-bgl, rec104Δ::KanMX*
SKY7405	*MATα,ho::LYS2, lys2, leu2::hisG, trp1::hisG, arg4-nsp, rec104Δ::KanMX*

Table S3. Plasmids

Plasmid	Description	Reference
pCCB586	*SPO11* from *S. cerevisiae* (SK1 strain) in pFastBac1	Claeys Bouuaert et al., 2021
pCCB587	*SKI8* from *S. cerevisiae* (SK1 strain) in pFastBac1	Claeys Bouuaert et al., 2021
pCCB588	*REC102* from *S. cerevisiae* (SK1 strain) in pFastBac1	Claeys Bouuaert et al., 2021
pCCB589	*REC104* from *S. cerevisiae* (SK1 strain) in pFastBac1	Claeys Bouuaert et al., 2021
pSK275	LexA empty Y2H vector	Arora et al., 2004
pSK276	Gal4AD empty Y2H vector	Arora et al., 2004
pSK282	LexA-Rec102 Y2H vector (pCA1-Rec102)	Maleki et al., 2007
pSK293	Rec104-LexA Y2H vector	Arora et al., 2004
pSK305	Gal4AD-Spo11 Y2H vector	Arora et al., 2004
pSK310	Gal4AD-Rec104 Y2H vector	Arora et al., 2004
pZZy1	pSK305 with *spo11-L53A*	This study
pZZy2	pSK305 with *spo11-L60A*	This study
pZZy3	pSK305 with *spo11-F91A*	This study
pZZy4	pSK305 with *spo11-L105A*	This study
pZZy5	pSK305 with *spo11-L112A*	This study
pZZy6	pSK305 with *spo11-L93A*	This study
pZZy7	pSK305 with *spo11-N94A*	This study
pZZy8	pSK305 with *spo11-G95A*	This study
pZZy9	pSK305 with *spo11-L98A*	This study
pZZy10	pSK305 with *spo11-F103A*	This study
pZZy11	pSK305 with *spo11-L3A*	This study
pZZy12	pSK305 with *spo11-R6A*	This study
pZZy13	pSK305 with *spo11-R7A*	This study
pZZy14	pSK305 with *spo11-R7D*	This study
pZZy15	pSK305 with *spo11-L16A*	This study
pZZy16	pSK305 with *spo11-V17A*	This study
pZZy17	pSK305 with *spo11-L20A*	This study
pZZy18	pSK282 with *rec102-L207A*	This study
pZZy19	pSK282 with *rec102-I195A*	This study
pZZy20	pSK282 with *rec102-L198A*	This study
pZZy21	pSK282 with *rec102-R199A*	This study

pZZy22	pSK282 with *rec102-W202A*	This study
pZZy23	pSK282 with *rec102-S233A*	This study
pZZy24	pSK282 with *rec102-I249A*	This study
pZZy25	pSK282 with *rec102-L8A*	This study
pZZy26	pSK282 with *rec102-V10A*	This study
pZZy27	pSK282 with *rec102-L179A*	This study
pZZy28	pSK282 with *rec102-S182A*	This study
pZZy29	pSK282 with *rec102-F11A*	This study
pZZy30	pSK282 with *rec102-F156A*	This study
pZZy31	pSK310 with *rec104-I11A*	This study
pZZy32	pSK310 with *rec104-T12A*	This study
pZZy33	pSK310 with *rec104-F17A*	This study
pZZy34	pSK310 with *rec104-L18A*	This study
pZZy35	pSK310 with *rec104-Y21A*	This study
pZZy36	pSK310 with *rec104-F31A*	This study
pZZy37	pSK310 with *rec104-L33A*	This study
pZZy38	pSK310 with *rec104-V50A*	This study
pZZy39	pSK310 with *rec104-I53A*	This study
pZZy40	pSK293 with *rec104-D16A*	This study
pZZy41	pSK293 with *rec104-D16R*	This study
pZZy42	pSK293 with *rec104-Y21A*	This study
pZZy43	pSK305 with *spo11-R13A*	This study
pZZy44	pSK305 with *spo11-K8A*	This study
pZZy45	pSK282 with *rec102-Q183A*	This study
pZZy46	pSK305 with *spo11-Q012A*	This study
pZZy47	pSK282 with *rec102-Q239A*	This study
pZZy48	pSK293 with *rec104-F22A*	This study
pZZy49	pSK293 with *rec104-Q20A*	This study
pZZy50	pSK310 with *rec104-K10A*	This study
